# Comprehensive pan-cancer investigation of carnosine dipeptidase 1 and its prospective prognostic significance in hepatocellular carcinoma

**DOI:** 10.1515/med-2024-0982

**Published:** 2024-06-14

**Authors:** Xiao-Wen Huang, Yan Li, Li-Na Jiang, Bo-Kang Zhao, Yi-Si Liu, Chun Chen, Dan Zhao, Xue-Li Zhang, Mei-Ling Li, Yi-Yun Jiang, Shu-Hong Liu, Li Zhu, Jing-Min Zhao

**Affiliations:** Medical School of Chinese PLA, Beijing, China; Department of Pathology and Hepatology, The Fifth Medical Center of Chinese PLA General Hospital, Beijing, 100039, China; Department of Hepatology, Center of Infectious Diseases and Pathogen Biology, The First Hospital of Jilin University, Changchun, China; First Department of Liver Disease Center, Beijing Youan Hospital, Capital Medical University, Beijing, China; Senior Department of Hepatology, The Fifth Medical Center of Chinese PLA General Hospital, Beijing, China

**Keywords:** carnosine dipeptidase 1, GEO, pan-cancer, hepatocellular carcinoma, prognosis, biomarker

## Abstract

Carnosine dipeptidase 1 (CNDP1), an enzyme integral to the hydrolysis of dipeptides containing histidine, plays an indispensable role in myriad physiological processes, including hydrolysis of proteins, maturation of specific biochemical functionalities within proteins, tissue regeneration, and regulation of cell cycle. However, the implications of CNDP1 in oncogenesis and its prognostic value are not yet fully elucidated. Initially, we procured the GSE40367 dataset from the Gene Expression Omnibus and established a protein–protein interaction network. Thereafter, we conducted functional and pathway enrichment analyses utilizing GO, KEGG, and GSEA. Moreover, we undertook an association analysis concerning the expression of CNDP1 with immune infiltration, along with survival analysis across various cancers and specifically in hepatocellular carcinoma (HCC). Our study uncovered a total of 2,248 differentially expressed genes, with a down-regulation of CNDP1 in HCC and other cancers. Our explorations into the relationship between CNDP1 and immune infiltration disclosed a negative correlation between CNDP1 expression and the presence of immune cells in HCC. Survival analyses revealed that diminished expression of CNDP1 correlates with an adverse prognosis in HCC and several other types of cancer. These observations intimate that CNDP1 holds promise as a novel prognostic biomarker for both pan-cancer and HCC.

## 1 Introduction

According to the most recent data from the World Health Organization (WHO), approximately 1,996 million new cancer cases were recorded globally in 2022, underscoring cancer’s persistent status as a formidable public health challenge. Notably, hepatocellular carcinoma (HCC) is ranked as the eighth most common cancer worldwide and emerges as the third leading cause of cancer-related mortality [1]. Given the complex mechanisms of tumorigenesis, it is imperative to undertake a thorough investigation of gene expression across diverse cancer types to discern their correlation with clinical outcomes and the underlying molecular mechanisms. Surgical resection and liver transplantation represent therapeutic strategies aimed at curing HCC [2]. Despite these interventions, a considerable proportion of individuals diagnosed with HCC encounter the recurrence of the tumor within a 5-year timeframe [3]. Consequently, there is an urgent need to develop innovative prognostic assessment tools to better predict the clinical outlook of individuals diagnosed with HCC and across various cancers. The establishment of a prognostic model to predict survival probabilities and stratify patient outcomes is of paramount importance.

Numerous biomarkers, including alpha-fetoprotein (AFP), AFP-L3, and DCP; as well as GPC3, HSP70, and SCCA, have been extensively explored as potential indicators for the diagnosis and prognosis of HCC [4–7]. Additionally, a plethora of molecular mechanisms are implicated in the onset and advancement of HCC, involving mutations in genes like TP53, CTNNB1, and AXIN1, and the disruption of signaling pathways including the Wnt/β-catenin pathway and the PI3K/AKT/mTOR pathway [8–10]. A deeper comprehension of these molecular dynamics is instrumental in forging pathways toward the creation of targeted treatments for HCC.

Carnosine dipeptidase 1 (CNDP1) is a gene responsible for encoding proteins, situated on chromosome 18q22.3. The resultant protein, CNDP1, with a molecular weight of 56.8 kDa, is predominantly expressed in cerebral tissues and constitutes a homodimeric dipeptidase, recognized as human carnosinase [11]. The gene features a trinucleotide (CTG) repeat length polymorphism within its coding sequence [12]. CNDP1 functions both as a secreted and intracellular entity, localized externally to cell membranes [11].

Recent findings indicate that CNDP1 is a molecule conspicuously downregulated in various cancer forms, including HCC. As a proteolytic enzyme, it specializes in cleaving histidine-containing dipeptides and is integral to numerous physiological functions, including protein degradation, maturation of specific proteins, tissue restoration, and cellular cycle regulation [13].

Previous studies have noted certain correlations between CNDP1 and specific cancer types [14–17]. However, these correlations have not been definitively established across all forms of cancer. The link between CNDP1 and cancer seems not to be universally applicable, but rather appears to be contingent upon a variety of factors, including the type of tumor, its developmental stage, and individual patient variations. Consequently, it is essential that we conduct an exhaustive investigation into the role and efficacy of CNDP1 in a pan-cancer context. Only through such a meticulous approach can we accurately determine whether CNDP1 presents a viable new target for cancer therapy, thus offering renewed hope to those afflicted by this disease.

In this study, we scrutinized GSE40367 dataset acquired from Gene Expression Omnibus (GEO). We identified prevalent differentially expressed genes (DEGs) within the dataset and executed protein–protein interaction (PPI), as well as functional and pathway enrichment analyses. Remarkably, CNDP1 was discerned as one of the top three genes with down-regulated expression, showing significant differential alterations, yet its association with HCC had not been documented in previous research. Consequently, CNDP1 was designated as the focal gene for this study. Our results suggest that the expression of CNDP1 is intricately connected to the immune response and holds considerable promise as a valuable prognostic biomarker for various malignancies, including HCC.

## 2 Materials and methods

### 2.1 Patients of study

Between October 2021 and June 2022, a cohort of 75 patients diagnosed with HCC was enrolled at the Fifth Medical Center of Chinese PLA General Hospital. The diagnosis adhered to the 2019 WHO classification guidelines for digestive system tumors [18], and were corroborated by two independent pathologists.

### 2.2 Expression profile dataset selection

Datasets pertinent to mRNA associated with HCC were meticulously selected through an exhaustive search of the publicly available GEO dataset portal on NCBI (https://www.ncbi.nlm.nih.gov/geo/), using the search terms “HCC” and “Homo sapiens.” Within the array of datasets, GSE40367, contributed by Roessler et al., was pinpointed. This particular dataset comprises 61 samples, encompassing colon adenocarcinoma, liver hemangioma, HCC, cholangiocarcinomas, and angiosarcoma. For our analysis, we selected five liver hemangioma samples to serve as normal liver controls alongside 32 HCC samples. The GSE40367 dataset is predicated on the GPL570 platform, employing the Affymetrix Human Genome U133 Plus 2.0 Array [19].

### 2.3 DEGs identification

Using the limma software package, we conducted an analysis on datasetGSE40367 to identify DEGs. The methodology incorporated adjusted *P* (adj.*P*) alongside the Benjamini and Hochberg false discovery rate, thus ensuring a meticulous balance between the identification of statistically significant genes and the mitigation of false positives. Probe sets devoid of gene symbols, or those corresponding to multiple gene symbols, were either excluded or consolidated, respectively. DEGs were ascertained using a threshold of |log2FC| >1.0 and adj.*P* < 0.05, signifying a minimum two-fold change in expression levels between the compared groups, a robust indicator of significant differential expression unlikely to be attributable to mere chance.

### 2.4 PPI network construction

To elucidate the gene connections, the DEGs were incorporated into the Search Tool for the Retrieval of Interacting Genes [STRING (version 12.0)] online platform (http://string-db.org) to construct the PPI network, adhering to stringent criteria (minimum requisite interaction score: highest confidence 0.900, *k*-means clustering: number of clusters 3). In this study, STRING facilitated the analysis of the PPI of DEGs among the top 100 in differential expression magnitude. Following this, leveraging the interaction data, the network was crafted and depicted using Cytoscape software (version 3.9.1). A roster of protein intermediaries was procured and subsequently, Cytoscape was employed to delineate and scrutinize PPI networks, considering an interaction score of no less than 0.4 as significant. The PPI network was delineated by filtering pivotal protein expression molecules via the Minimal Common Oncology Data Elements of Cytoscape plug-in. Additionally, the CytoNCA plug-in, adopting a centrality-focused methodology, was utilized to identify hub genes within the PPI networks. All targets were methodically arranged into circles, with a high centrality value denoting a paramount role within the network.

### 2.5 Functional and pathway enrichment analysis

DEGs were analyzed for Gene Ontology (GO) and Kyoto Encyclopedia of Genes and Genomes (KEGG) pathway enrichments using the clusterProfiler package in R, as facilitated by the bioinformatics platform (https://www.bioinformatics.com.cn) [20]. The GO enrichment analysis is divided into three categories: biological processes (BP), cellular components (CC), and molecular functions (MF). Additionally, gene set enrichment analysis was performed utilizing the R packages “clusterProfiler” and “GSVA,” with the selected gene set annotated as (h.all.v7.2.symbols.gmt). The Normalized Enrichment Score (NES) was determined following 1,000 permutations. A gene set was deemed significantly enriched if it met the criteria of |NES| > 1, *P* < 0.05, and a false discovery rate <0.25. The results were elegantly visualized in a bubble plot created with the “ggplot2” R package.

### 2.6 Analysis of CNDP1 expression

The Tumor Immune Estimation Resource 2.0 (TIMER2.0, http://timer.cistrome.org/) leverages high-throughput sequencing data to scrutinize immune cell infiltration within tumor specimens, juxtaposing these findings with those from control normal tissues. The Gene Expression Profilling Interactive Analysis 2 (GEPIA2, http://gepia2.cancer-pku.cn/#index) integrates cutting-edge cancer genomics data to facilitate efficient data mining and dynamic examination of gene expression profiles. Both TIMER2.0 and GEPIA2 were selected to investigate the variances in CNDP1 expression across diverse cancers, including HCC.

### 2.7 Immunohistochemical staining

The differential protein expression levels of CNDP1 in HCC and corresponding normal tissues were obtained from the Human Protein Atlas (HPA, https://www.proteinatlas.org/), encompassing both healthy and oncological tissue samples. These extensive expression profiles are discernible through the examination of tissue specimens.

### 2.8 Biochemical function, intracellular distribution, and structural information of CNDP1

The UniProt repository, an amalgamation of data from several esteemed databases, furnishes exhaustive details concerning the biochemical functionality, intracellular localization, and structural attributes of proteins. Utilizing the terms “CNDP1” and “HUMAN,” we pinpointed the protein of interest (Q96KN2 CNDP1 HUMAN). The protein annotation information provided by UniProt, including function, subcellular localization, and structure, is crucial for gaining a deeper understanding of the biological function of CNDP1 and the potential mechanism of its action.

### 2.9 Association analysis of CNDP1 expression with immune cell infiltration in HCC

Tumor-infiltrating lymphocytes have been recognized as independent prognostic indicators for both the status of sentinel lymph nodes and the overall survival rate in cancer patients. The TIMER2.0 database was employed to ascertain the relationship between immune infiltration and CNDP1 expression in HCC [21]. *P* < 0.05 was considered statistically significant. After adjusting for tumor purity using the Spearman correlation coefficient, a *P* < 0.05 and a Rho > 0 denoted a positive correlation, whereas a *P* < 0.05 and a Rho < 0 indicated a negative correlation.

### 2.10 Gene mutation, immuno-infiltration, and methylation analysis in pan-cancer

Utilizing the cBioPortal database (http://www.cbioportal.org/) and the Gene Set Cancer Analysis platform (http://bioinfo.life.hust.edu.cn/GSCA/#/), we conducted an in-depth analysis of the expression of CNDP1, emphasizing variations in gene copy number and methylation processes. The UALCAN database (http://ualcan.path.uab.edu) was instrumental in providing data concerning DNA methylation levels within the promoter of the CNDP1. For the examination of immune correlations, we employed the sophisticated EPIC [22] and CIBERSORT [23] algorithms to calculate the Spearman’s correlation coefficient, delineating the relationship between CNDP1 expression and immune cell infiltration across various tumors, depicted via a comprehensive heat map. Furthermore, we explored the association between CNDP1 expression and tumor mutational burden (TMB) within the TCGA cohort, employing the “maftools” R package for analysis. The correlation of CNDP1 expression with TMB across different cancer types was meticulously assessed using the Spearman method, with findings eloquently presented through both heat map and radar map visualizations.

### 2.11 Prognostic analysis

In this study, we partitioned the survival data of distinct cancers from the TCGA database into cohorts with high and low CNDP1 expression based on median gene expression levels. To ascertain the prognostic relevance of CNDP1 in cancer, we conducted a Kaplan–Meier survival analysis. We employed four clinical metrics – overall survival (OS), disease-free survival (DFS), disease-specific survival (DSS), progression-free interval (PFI), and disease-free interval (DFI)  – to explore the association between CNDP1 expression levels and patient prognoses. Forest plots for Cox regression analysis were generated using the “forestplot” and “survival” packages in R. We further investigated the correlation between the variation in CNDP1 expression and the prognosis of HCC patients across varying tumor microenvironments. Hazard ratios with 95% confidence intervals (CI) were calculated, along with log-rank *P* values. A threshold level of *P* < 0.05 was considered statistically significant.

### 2.12 Laboratory and MRI as well as histopathologic examination from our own samples

All preoperative routine examination parameters, encompassing laboratory assessments and MRI, were meticulously gathered from the electronic medical record system of the hospital. The imaging diagnostic outcomes were expertly analyzed by two distinguished senior radiologists, whereas the histopathological assessments were performed by two seasoned pathologists. Serum CNDP1 concentrations were quantified employing an enzyme-linked immunosorbent assay (ELISA). For the purpose of measuring serum CNDP1 levels, the ELISA Kit (EK1957, BOSTER, Wuhan, China) was utilized.

### 2.13 Statistical analysis

To evaluate statistical significance, the expression levels of CNDP1 in tumor versus normal tissues were compared employing *T*-tests or Wilcoxon rank sum tests. The association between variables was scrutinized using either Spearman or Pearson correlation tests. The Kaplan–Meier method, log-rank test, and Cox proportional hazards regression model facilitated the analysis of pan-cancer survival rates. Both univariate and multivariate logistic regression analyses were employed to construct a prognostic model for HCC. Statistical significance was deemed established at *P* < 0.05 for most analyses. The analytical procedures and the creation of the nomogram were performed using SPSS, version 21.0, and R, version 4.2.2.


**Ethical approval:** This study received ethical approval from the Institutional Ethics Committee of the Fifth Medical Center of Chinese PLA General Hospital.

## 3 Results

### 3.1 Identification of DEGs in GSE40367 and PPI network construction of DEGs

**Figure 1:**
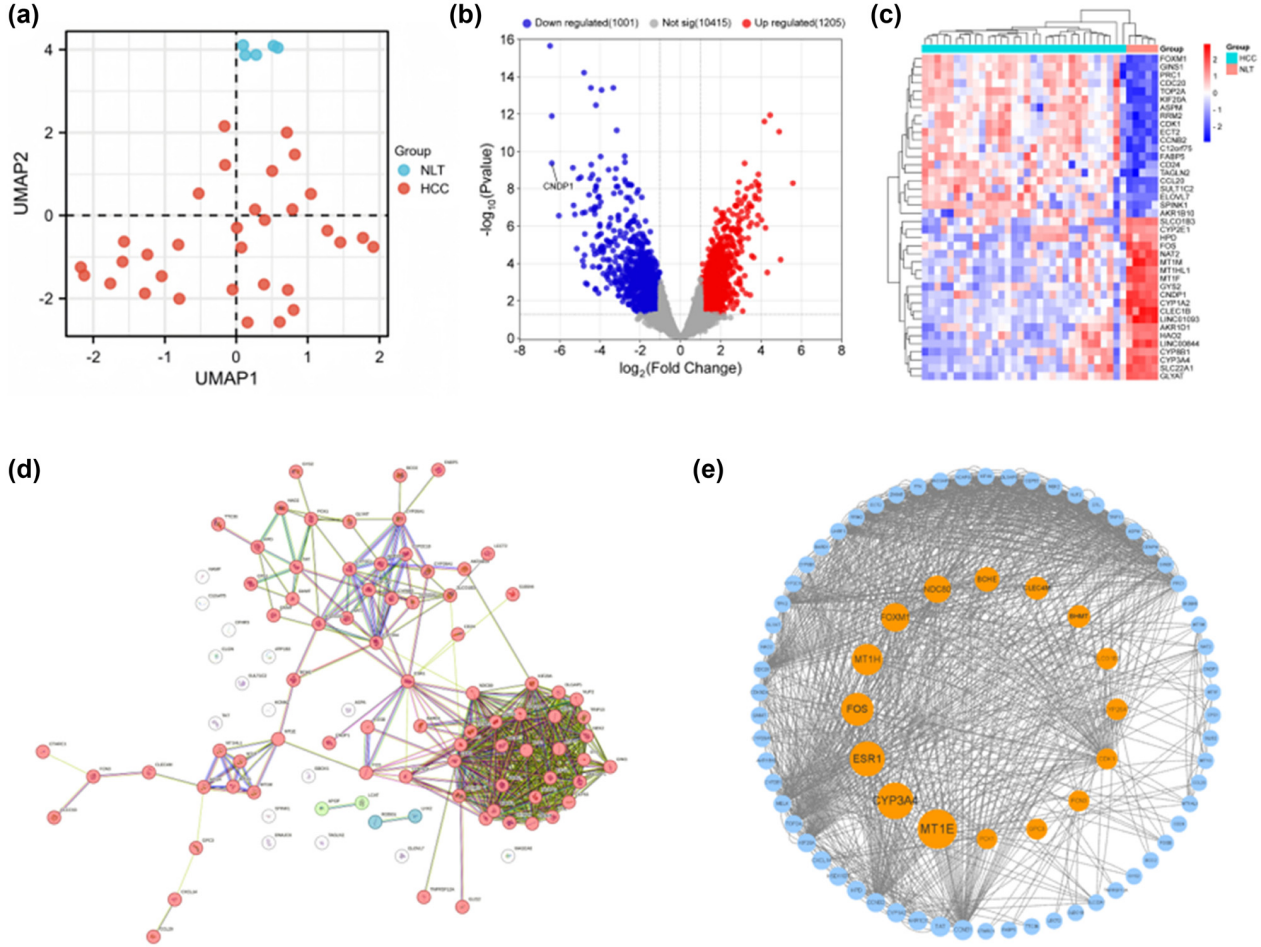
Transcriptome analysis of GSE40367 microarray. (a) UMAP score plot of HCC group and normal liver tissue in GSE40367. (b) Volcano plot of DEGs in GSE40367. Red dots indicate high-expressed genes, blue dots indicate low-expressed genes, and gray dots indicate genes that are undifferentially expressed according to *P* < 0.05 and |log2FC| > 1.0. (c) Heatmap of DEGs shows hierarchical cluster analysis of gene transcriptional changes in two groups. HCC, hepatocellular carcinoma; NLT, normal liver tissue. (d) PPI network of top 50 up-regulated targets and the top 50 down-regulated targets according to the STRING database. The network’s nodes symbolize proteins, the edges represent protein–protein associations. (e) The protein interaction network derived from the PPI analysis using the cytoNCA plugin in Cytoscape, features circular shapes representing the proteins and lines delineating the interactions between them.

We conducted an exhaustive analysis of GEO datasets pertaining to patients diagnosed with HCC. Consequently, GSE40367 was identified as the dataset of choice. The information of the samples contained within this dataset facilitated the identification of DEGs (healthy controls [5 samples] versus HCC patients [32 samples]). Employing the limma package, we discerned 1,121 upregulated and 1,127 downregulated DEGs in GSE40367 following Log2 transformation (Figure 1a–c). The top 20 genes exhibiting significant variations are enumerated in Table 1.

**Table 1:** Top 20 genes with different changes

ID_REF	Gene symbol	AveExpr	*P* value	Adj.*P* Val	logFC
1559573_at	LINC01093	5.3662	2.23 × 10^−16^	2.81 × 10^−12^	−6.4809
223699_at	CNDP1	6.085	4.19 × 10^−10^	3.37 × 10^−7^	−6.3928
207608_x_at	CYP1A2	7.6481	1.27 × 10^−12^	2.00 × 10^−9^	−6.3917
207201_s_at	SLC22A1	6.5374	2.82 × 10^−7^	3.71 × 10^−5^	−6.0425
205476_at	CCL20	8.1218	5.12 × 10^−9^	2.02 × 10^−6^	5.5766
206797_at	NAT2	5.7728	7.40 × 10^−10^	5.19 × 10^−7^	−5.3452
205998_x_at	CYP3A4	7.8762	7.93 × 10^−8^	1.45 × 10^−5^	−5.3105
206930_at	GLYAT	6.6243	2.42 × 10^−7^	3.36 × 10^−5^	−5.2457
206354_at	SLCO1B3	6.9485	1.64 × 10^−5^	0.00088778	−5.1534
217165_x_at	MT1F	8.9417	2.92 × 10^−9^	1.42 × 10^−6^	−5.0585
206239_s_at	SPINK1	9.9658	6.38 × 10^−5^	0.0023602	4.968
217546_at	MT1M	4.9946	2.36 × 10^−9^	1.30 × 10^−6^	−4.952
201890_at	RRM2	8.9564	8.67 × 10^−12^	9.95 × 10^−9^	4.8941
230577_at	LINC00844	5.2931	2.01 × 10^−7^	2.89 × 10^−5^	−4.859
232494_at	CYP8B1	8.793	7.30 × 10^−5^	0.0025834	−4.8576
220801_s_at	HAO2	7.4492	0.0001387	0.0040821	−4.8079
220496_at	CLEC1B	4.6874	6.04 × 10^−15^	3.81 × 10^−11^	−4.7912
207102_at	AKR1D1	7.8868	3.64 × 10^−5^	0.0015746	−4.7662
214621_at	GYS2	7.2327	2.69 × 10^−6^	0.00021873	−4.7534
209189_at	FOS	6.2349	1.86 × 10^−7^	2.77 × 10^−5^	−4.7391

AveExpr: the average expression of the gene in all samples; logFC: takes log2 for HCC/normal NLT. HCC, hepatocellular carcinoma; NLT, normal liver tissue.

Within the constructed PPI network, which comprised 97 nodes linked by 505 edges, a significant enrichment was observed (*P* < 1.0 × 10^−16^) (Figure 1d). The network visualization was facilitated using Cytoscape software (Figure 1e). Employing the median center algorithm, we identified 16 genes of paramount importance, suggesting their potential as pivotal genetic determinants: MT1E, CYP3A4, ESR1, FOS, MT1H, FOXM1, NDC80, BCHE, CLEC4M, BHMT, SLCO1B3, CYP26A1, CDK1, FNC3, GPC3, and PCK1.

### 3.2 Functional and pathway enrichment analysis

**Figure 2:**
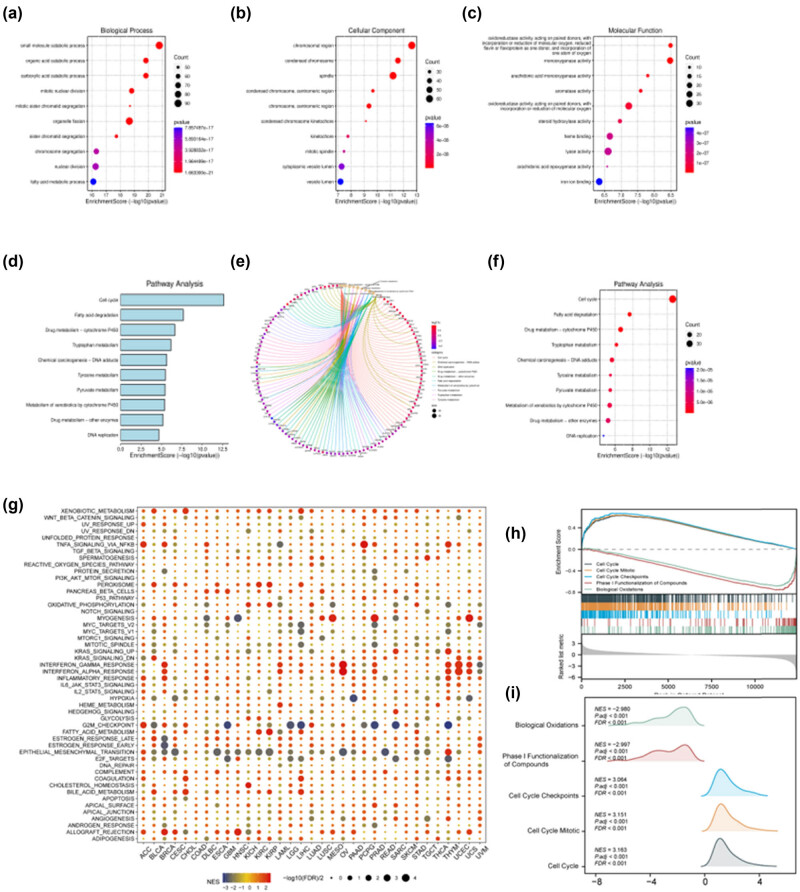
Enrichment pathway analysis of DEGs in GSE40367 microarray. (a)–(c) Bubble plot of GO enrichment analysis results. *X*-axis represents the percentage of genes associated with each functional term. *Y*-axis represents the annotated terms of gene enrichment. The size of each circle corresponds to the number of genes, with larger circles representing a greater gene count. The color of the circles reflects the adjusted *P* value. (d)–(f) Bar, chord, and bubble plot of KEGG enrichment analysis results. In the bar plot, *X*-axis represents the ES of protein enrichment, *Y*-axis represents the top 10 pathways ranked by the degree value of KEGG signaling pathways. In bubble plot, *X*-axis represents the percentage of genes associated with each functional term. *Y*-axis represents the annotated terms of gene enrichment. The size of each circle corresponds to the number of genes, with larger circles indicating a greater gene count. The color of the circles reflects the adjusted *P* value. In the chord plot, the line segments connect genes and enrichment pathways, with different colors representing distinct enrichment pathways. The size of the circles corresponds to the number of connected line segments; larger circles indicate a greater number of connected genes and pathways. Yellow circles represent pathways, other circles represent genes. (g)–(i) GSEA analysis of GSE40367. (g) Functional analysis of CNDP1 in pan-cancer. The heat map utilizes color-coding to visually represent the degree of gene enrichment, red signifies higher ESs and blue signifies lower ESs. Each row corresponds to a distinct gene set, and each column corresponds to a distinct type of cancer. (h) GSEA visual analysis shows the concentration of gene sets in the sorted list. (i) GSEA ridgeplot illustrates how the ES of a gene set changes with the sorting of the gene list. The *X*-axis represents the sequenced gene list, while the *Y*-axis represents the ES. The curve (or “mountain”) in the figure demonstrates how the ES of a particular gene set changes cumulatively as the list of genes is traversed. The peak ES indicates the region of enrichment for the gene set in the list. DEGs, differentially expressed genes; ES, enrichment score.

To elucidate the roles of DEGs in GSE40367, we conducted a GO enrichment analysis. The findings revealed that the BP predominantly encompass the catabolism of small molecules, organelle fission, chromosome segregation, nuclear division, and the metabolic processing of fatty acids. CC functions were primarily associated with chromosomal regions, condensed chromosomes, spindles, chromosomes, centromeric regions, cytoplasmic vesicle lumens, and vesicle lumens. MF categories notably included activities including monooxygenase, oxidoreductase acting on paired donors with the incorporation or reduction of molecular oxygen, heme binding, lyase activity, and iron ion binding (Figure 2a–c).

Subsequent to our analysis, the KEGG enrichment yielded the following insights: cell cycle, fatty acid degradation, drug metabolism via cytochrome P450, tryptophan metabolism, chemical carcinogenesis through DNA adducts, tyrosine metabolism, pyruvate metabolism, metabolism of xenobiotics by cytochrome P450, drug metabolism by other enzymes, and DNA replication (Figure 2d–f).

Furthermore, we investigated potential signaling pathways through which aberrant CNDP1 expression influences functional states across 33 distinct cancer types via GSEA. We noted a marked enrichment in several signaling pathways, including epithelial–mesenchymal transition, G2/M checkpoint, xenobiotic metabolism, and immune-related pathways such as IFN-α response, IFN-γ response, and TNFα signaling via NFκB. Additionally, the predominant pathways associated with GSEA enrichment encompass biological oxidations, phase I functionalization of compounds, and various cell cycle processes like checkpoints and mitotic events (Figure 2g–i). The GO–KEGG analysis results for DEGs in GSE40367 and the CNDP1 gene set enrichment are concisely presented in Table 2.

**Table 2:** Top five GO–KEGG enrichment analysis results

Ontology	ID	Description	Gene ratio	BgRatio	*P* value	*P*.adjust	*q* value
BP	GO:0044282	Small molecule catabolic process	90/1249	452/18866	1.66 × 10^−21^	9.38 × 10^−18^	8.15 × 10^−18^
BP	GO:0016054	Organic acid catabolic process	67/1249	282/18866	1.47 × 10^−20^	2.76 × 10^−17^	2.40 × 10^−17^
BP	GO:0046395	Carboxylic acid catabolic process	67/1249	282/18866	1.47 × 10^−20^	2.76 × 10^−17^	2.40 × 10^−17^
BP	GO:0140014	Mitotic nuclear division	66/1249	286/18866	1.50 × 10^−19^	2.06 × 10^−16^	1.79 × 10^−16^
BP	GO:0000070	Mitotic sister chromatid segregation	48/1249	161/18866	1.98 × 10^−19^	2.06 × 10^−16^	1.79 × 10^−16^
CC	GO:0098687	Chromosomal region	63/1290	350/19559	2.38 × 10^−13^	1.50 × 10^−10^	1.32 × 10^−10^
CC	GO:0000793	Condensed chromosome	46/1290	222/19559	2.74 × 10^−12^	8.61 × 10^−10^	7.57 × 10^−10^
CC	GO:0005819	Spindle	62/1290	367/19559	6.46 × 10^−12^	1.35 × 10^−9^	1.19 × 10^−9^
CC	GO:0000779	Condensed chromosome, centromeric region	30/1290	122/19559	2.20 × 10^−10^	3.46 × 10^−8^	3.05 × 10^−8^
CC	GO:0000775	Chromosome, centromeric region	39/1290	196/19559	4.39 × 10^−10^	5.52 × 10^−8^	4.86 × 10^−8^
MF	GO:0016712	Oxidoreductase activity, acting on paired donors, with incorporation or reduction of molecular oxygen, reduced flavin or flavoprotein as one donor, and incorporation of one atom of oxygen	15/1271	35/18352	3.23 × 10^−9^	1.67 × 10^−6^	1.43 × 10^−6^
MF	GO:0004497	Monooxygenase activity	26/1271	101/18352	3.33 × 10^−9^	1.67 × 10^−6^	1.43 × 10^−6^
MF	GO:0008391	Arachidonic acid monooxygenase activity	11/1271	20/18352	1.58 × 10^−8^	5.28 × 10^−6^	4.52 × 10^−6^
MF	GO:0070330	Aromatase activity	12/1271	25/18352	2.58 × 10^−8^	6.46 × 10^−6^	5.53 × 10^−6^
MF	GO:0016705	Oxidoreductase activity, acting on paired donors, with incorporation or reduction of molecular oxygen	32/1271	162/18352	5.91 × 10^−8^	1.18 × 10^−5^	1.01 × 10^−5^
KEGG	hsa04110	Cell cycle	39/689	127/8223	2.40 × 10^−13^	7.77 × 10^−11^	6.31 × 10^−11^
KEGG	hsa00071	Fatty acid degradation	17/689	43/8223	2.15 × 10^−8^	3.48 × 10^−6^	2.83 × 10^−6^
KEGG	hsa00982	Drug metabolism – cytochrome P450	21/689	72/8223	2.31 × 10^−7^	2.49 × 10^−5^	2.03 × 10^−5^
KEGG	hsa00380	Tryptophan metabolism	15/689	42/8223	6.99 × 10^−7^	5.66 × 10^−5^	4.60 × 10^−5^
KEGG	hsa05204	Chemical carcinogenesis – DNA adducts	19/689	69/8223	2.28 × 10^−6^	1.48 × 10^−4^	1.20 × 10^−4^

GO, Gene Ontology; KEGG, Kyoto Encyclopedia of Genes and Genomes; BP, biological process; CC, cellular component; MF, molecular function.

### 3.3 Expression analysis of CNDP1

**Figure 3:**
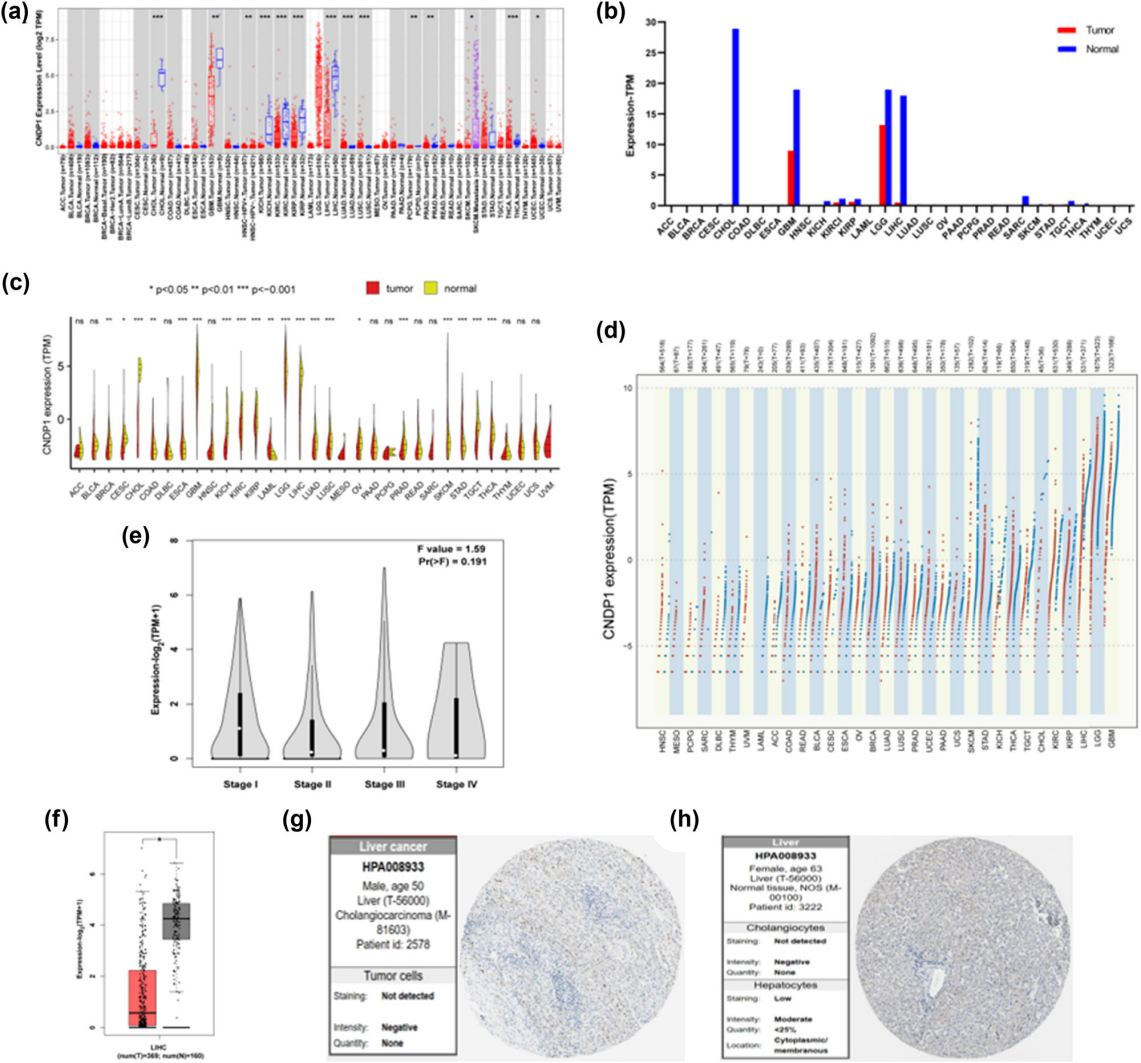
Differential expression of CNDP1. (a) *CNDP1* gene expression in different cancer types from TIMER2.0. Red dots represent the tumor group, blue dots represent the normal control group, and for cancer species with a control group, the background color is shown as gray. The statistical significance calculated by the Wilcoxon test is displayed above the bar chart. (b) *CNDP1* gene expression in different cancer types from GEPIA2. The red columns represent the tumor group and the blue columns represent the normal control group. (c) *CNDP1* gene expression in different cancer types from TCGA. The red areas represent the tumor group and the yellow areas represent the normal control group. (d) *CNDP1* gene expression in different cancer types from TCGA. The red dots represent the tumor group and the blue dots represent the normal control group. (e) *CNDP1* gene expression in different stages of HCC progression, derived from GEPIA2. (f) *CNDP1* gene expression in HCC tissues (LIHC) and normal liver tissues, derived from GEPIA2. (g) and (h) Expression of CNDP1 in HCC tissues and normal liver tissues at protein expression level, derived from the HPA database. CNDP1, carnosine dipeptidase 1; HCC, hepatocellular carcinoma (*, *P* < 0.05; **, *P* < 0.01; ***, *P* < 0.001).

We explored CNDP1 expression levels across diverse cancer types (Figure 3a–d). Utilizing the Kruskal–Wallis test, we assessed the variance in CNDP1 expression among different normal tissues. Data from TIMER2.0 reveal that in cholangiocarcinoma (CHOL), glioblastoma multiforme (GBM), head and neck squamous cell carcinoma (HNSC), kidney chromophobe (KICH), kidney renal clear cell carcinoma (KIRC), kidney renal papillary cell carcinoma (KIRP), liver hepatocellular carcinoma (LIHC), lung adenocarcinoma (LUAD), lung squamous cell carcinoma (LUSC), pheochromocytoma and paraganglioma (PCPG), prostate adenocarcinoma (PRAD), skin cutaneous melanoma (SKCM), thyroid carcinoma (THCA), and uterine corpus endometrial carcinoma (UCEC), the CNDP1 expression levels in tumor tissues were significantly lower than those in the corresponding normal tissues. This analysis underscores a prevalent pattern of diminished CNDP1 expression in solid tumors compared to normal counterparts.

We conducted an in-depth analysis concerning the expression of CNDP1 in HCC (LIHC) and different stages of HCC progression. Our findings disclosed a marked diminution in the expression levels of CNDP1 across 369 HCC specimens compared to 160 normal hepatic tissues (Figure 3f). Furthermore, it was observed that the expression of CNDP1 was preeminent during clinical stage I, diminished profoundly by clinical stage IV, and exhibited intermediate levels in clinical stages II and III (Figure 3e). The HPA database, an amalgamation of proteomic, transcriptomic, and systems biology data, corroborates these findings, indicating a reduced expression of CNDP1 protein in HCC relative to normal liver tissue (Figure 3g and h).

### 3.4 The subcellular location, function, and structural analysis

**Figure 4:**
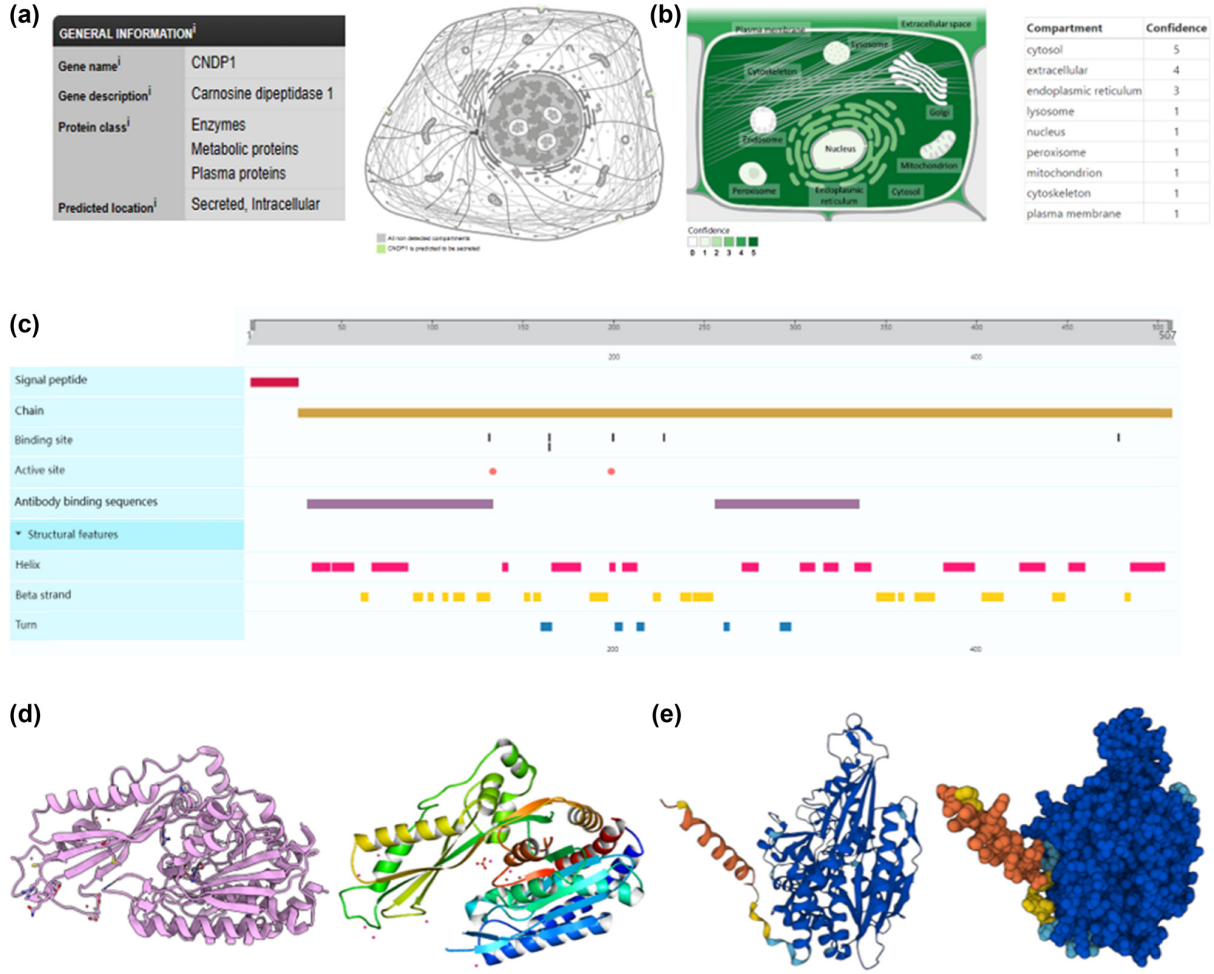
Intracellular distribution and structure of CNDP1. (a) Subcellular locations of CNDP1 protein from the HPA database. (b) Subcellular locations of CNDP1 protein from the GeneCards database. (c) Features of CNDP1 protein from the UniProt database. (d) Three-dimensional structures of CNDP1 protein from PDB (representative) (PDB: 3DLJ). (e) Three-dimensional structures of CNDP1 protein from AlphaFold (predicted) (AF-Q96KN2-F1). CNDP1, carnosine dipeptidase 1; HPA, Human Protein Atlas; PDB, Protein Data Bank.

According to the data sourced from the HPA and GeneCards databases, CNDP1 functions as both a secreted and intracellular protein, localized externally to cell membranes. This protein is distinguished by its capacity to catalyze the hydrolysis of the Xaa-His dipeptide via peptide bonds. It exhibits pronounced enzymatic activity toward carnosine (β-propionyl-l-histidine) and homocarnosine (β-propionyl-3-methyl-histidine) [11,24]. In its role as a catalyst, CNDP1 employs Zn^2+^ as a cofactor, coordinating two Zn^2+^ ions per subunit at the binding sites His132, Asp165, Glu200, and Asp228. Activation of CNDP1 is achieved through the binding of cadmium ions at residues Asp134 and Glu199, though it is impeded by the metal chelator 1,10-o-phenantrolin, which exhibits an inhibitory concentration of 50% (IC_50_) at 5 µM. The kinetic properties of CNDP1 are delineated in Table 3 [11,24]. Furthermore, CNDP1 presents two antibody-binding domains, specifically spanning residues 32–133 and 256–334. The protein is composed of 507 amino acids, the initial 26 of which constitute the signal peptide. The structural configuration of CNDP1, as documented in the Protein Data Bank (PDB: 3DLJ) and AlphaFold (https://alphafold.ebi.ac.uk/entry/Q96KN2, AF-Q96KN2-F1), comprises 18 beta strands (Figure 4).

**Table 3:** Kinetics of CNDP1

Km	Substrate	Temperature (°C)	Notes
1.27 μM	Carnosine	30	In the absence of cadmium ions
11 μM	Carnosine	30	In the presence of 200 µM cadmium ions
0.13 mM	Carnosine		
0.2 μM	Homocarnosine	30	In the absence of cadmium ions
1 μM	Homocarnosine	30	In the presence of 200 µM cadmium ions
8.7 mM	Homocarnosine		
*V*_max_			
8.5 μmol/min/mg			Toward carnosine
0.36 μmol/min/mg			Toward homocarnosine

CNDP1, carnosine dipeptidase 1; Km, Michaelis-Menten constant.

### 3.5 Immune cell infiltration in HCC

The tumor microenvironment constitutes an intricate, integrated system engendered by the interaction between neoplastic cells and the adjacent tissues and immune constituents. This milieu augments the proliferative, migratory, and immune evasion capabilities of the tumor cells, thus facilitating the onset and advancement of neoplastic conditions. Tumor-infiltrating lymphocytes have been identified as independent prognosticators of sentinel lymph node status and survival rates in oncological patients. Furthermore, analyses of immune infiltration have elucidated a correlation between CNDP1 expression and the level of immune infiltration in HCC.

**Figure 5:**
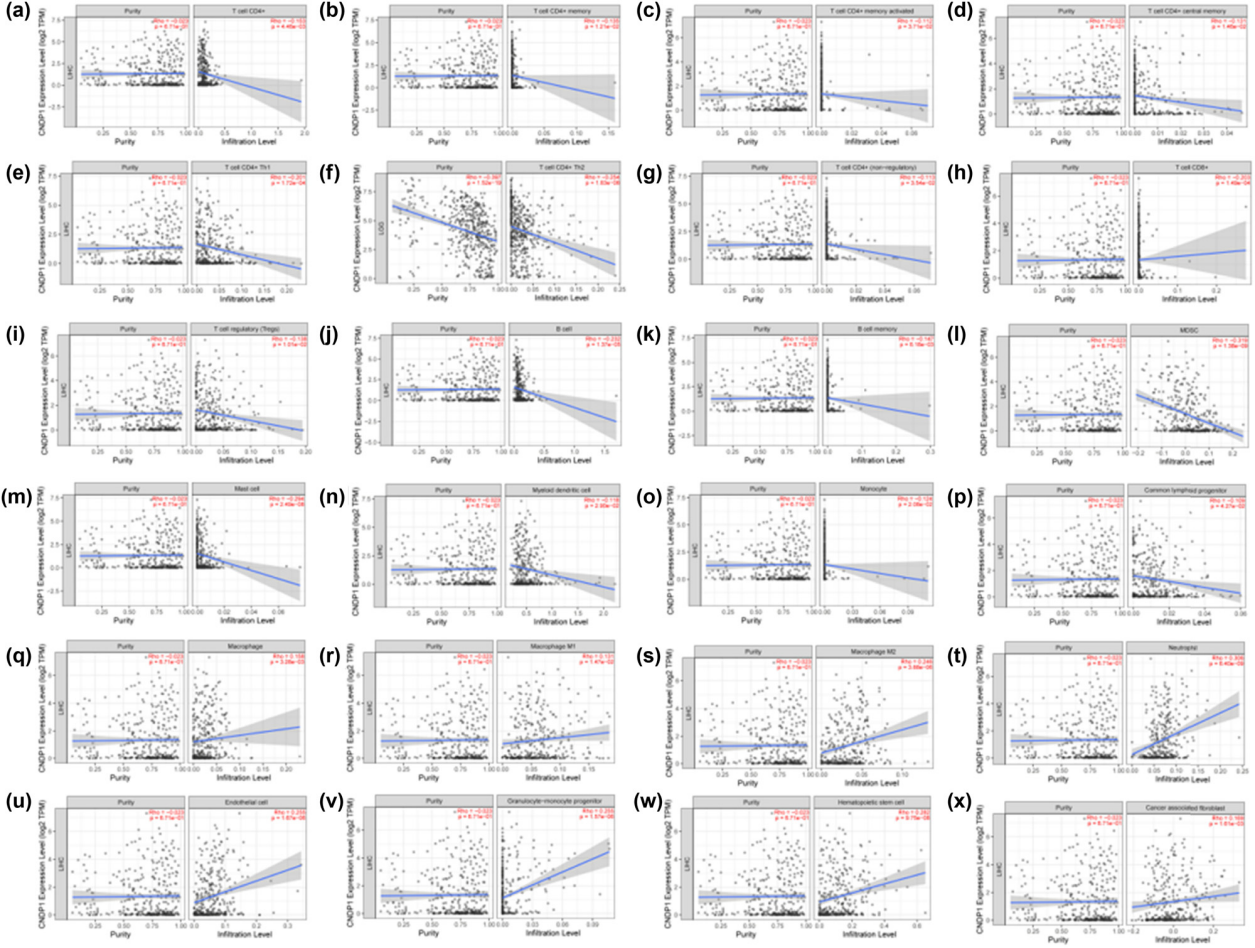
Correlation analysis between *CNDP1* expression and immune cell type in HCC. (a)–(x) Correlation analysis between *CNDP1* expression and different types of immune infiltrating cells in HCC. The Spearman correlation coefficient was utilized to calculate the relationship. CNDP1, carnosine dipeptidase 1; HCC, hepatocellular carcinoma*. P* < 0.05 and correlation coefficient (*R*) > 0 indicates a positive correlation; *P* < 0.05 and *R* < 0 indicates a negative correlation.

The expression of CNDP1 exhibited a negative correlation with various immune cells: CD4^+^ T cells, encompassing CD4^+^ T memory cells, CD4^+^ T memory activated cells, CD4^+^ T central memory cells, CD4^+^ Th1 cells, CD4^+^ Th2 cells, CD4^+^ T (non-regulatory) cells; CD8^+^ T cells; T regulatory cells; B cell, including B memory cells; myeloid derived suppressor cells (MDSC); mast cells; myeloid dendritic cells; monocyte; and common lymphoid progenitor cells. Conversely, the expression of CNDP1 was positively correlated with macrophage, including macrophage M1 cells and macrophage M2 cells; neutrophil; endothelial cells; granulocyte–monocyte progenitor; hematopoietic stem cell; and cancer-associated fibroblast (Figure 5). *P* < 0.05 was considered statistically significant.

### 3.6 Immunological landscape in pan-cancer

**Figure 6:**
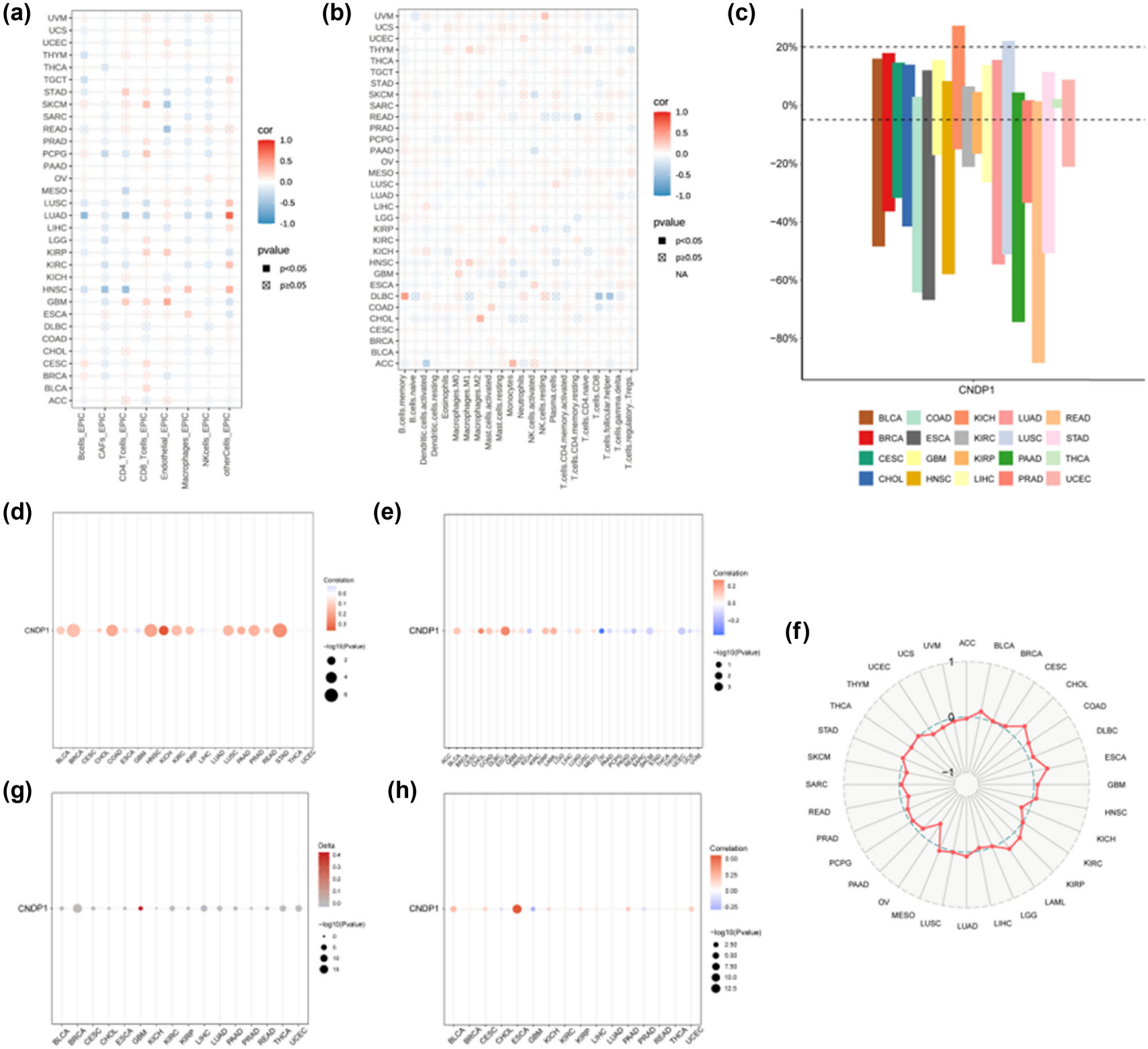
Immunological landscape in pan-cancer. (a) Analysis of immune cell infiltration levels and *CNDP1* expression from EPIC is presented. (b) Analysis of immune cell infiltration levels and *CNDP1* expression from CIBERSORT is provided. (c) The bar chart illustrates the rate of gene CNV for *CNDP1* in pan-cancer. (d) The bubble diagram demonstrates the relationship between *CNDP1* expression and CNV in pan-cancer. (e) The bubble map displays the correlation between *CNDP1* expression and TMB in pan-cancer. (f) The radar map showcases the association between *CNDP1* expression and TMB in pan-carcinoma. (g) The bubble map shows differences in promoter methylation of *CNDP1* between tumor and normal cells in pan-cancer. (h) The bubble plot illustrates the correlation between promoter methylation and expression of *CNDP1* in pan-cancer. CNDP1, carnosine dipeptidase 1; CNV, copy number variation; TMB, tumor mutational burden.

In this study, we employed the EPIC and CIBERSORT algorithms to explore the potential correlation between immune cell infiltration levels and CNDP1 expression across various cancer types in the TCGA dataset. The findings from the EPIC algorithms (Figure 6a) revealed a significant negative association between B cell immune infiltration and CNDP1 expression in thymoma (THYM), testicular germ cell tumors, stomach adenocarcinoma (STAD), LUSC, LUAD, and HNSC. Moreover, a positive correlation was observed between CD4 T cell infiltration and CNDP1 expression in STAD and GBM, while a negative correlation was noted between CD8 T cell infiltration and CNDP1 expression in LUSC and LUAD. The results obtained from the CIBERSORT algorithms are presented in Figure 6b.

We then extended our research to assess the copy number variations (CNV) for the CNDP1 gene in a pan-cancer context (Figure 6c). The findings disclosed a pronounced incidence of CNV in cancers such as KICH, THCA, GBM, and UCEC. Additionally, our examination of the relationship between CNV and CNDP1 expression in a pan-cancer overview (Figure 6d) revealed a distinct positive correlation between CNV levels and CNDP1 expression in multiple cancer types including bladder urothelial carcinoma (BLCA), breast invasive carcinoma, CHOL, colon adenocarcinoma (COAD), esophageal carcinoma (ESCA), HNSC, KICH, KIRC, KIRP, LUAD, LUSC, pancreatic adenocarcinoma (PAAD), PRAD, rectum adenocarcinoma (READ), and STAD. In contrast, GBM and HCC (LIHC) exhibited an inverse correlation between CNV and CNDP1 expression levels.

To explore the potential significance of CNDP1 in predicting the effectiveness of immune checkpoint inhibitor treatment, we examined the relationship between CNDP1 expression levels and TMB, a well-established biomarker for immunotherapy prediction. Our findings revealed a positive correlation between CNDP1 expressions in BLCA, CHOL, COAD, ESCA, HNSC, KIRP, acute myeloid leukemia, and LUAD with TMB values. Conversely, we observed a negative correlation between CNDP1 expressions in KICH, LIHC, ovarian serous cystadenocarcinoma (OV), PAAD, PRAD, READ, SKCM, and UCEC with TMB (Figure 6e and f).

Furthermore, elevated promoter methylation levels of CNDP1 were identified in GBM compared to other cancer types (Figure 6g). Additionally, the analysis of tumor and normal promoter methylation of CNDP1 across various cancers indicated a negative correlation between CNDP1 expression and CHOL, GBM, and PRAD; as well as a positive correlation with BLCA, cervical squamous cell carcinoma, endocervical adenocarcinoma, ESCA, KICH, KIRP, LIHC, PAAD, and UCEC (Figure 6h).

### 3.7 Prognostic analysis

**Figure 7:**
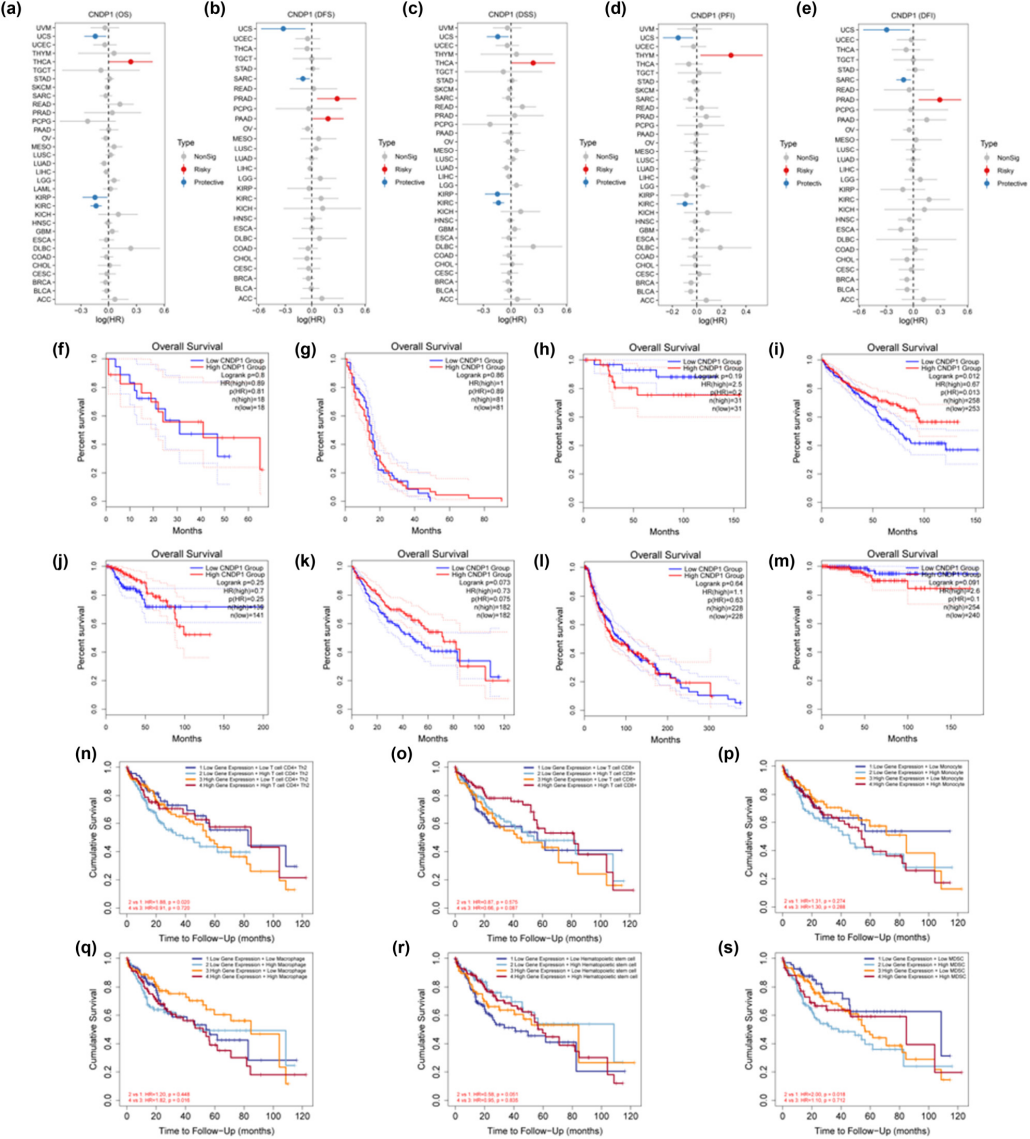
Correlation between *CNDP1* expression and prognosis in pan-cancer and HCC. (a)–(e) Correlation between *CNDP1* expression and OS, DFS, DSS, PFI, DFI in pan-cancer. (f)–(m) KM survival curves of high and low *CNDP1* expression in CHOL, GBM, KICH, KIRC, KIRP, LIHC(HCC), SKCM, and THCA patients. (n)–(s) KM curves of low and high *CNDP1* expression with CD4^+^ Th2 cells, CD8^+^ T cells, monocytes, macrophages, hematopoietic stem cells, and MDSC. CNDP1, carnosine dipeptidase 1; HCC, hepatocellular carcinoma; OS, overall survival; DFS, disease-free survival; DSS, disease-specific survival; PFI, progression-free interval; DFI, disease-free interval; CHOL, cholangiocarcinoma; GBM, glioblastoma multiforme; KICH, kidney chromophobe; KIRC, kidney renal clear cell carcinoma; KIRP, kidney renal papillary cell carcinoma; LIHC, liver hepatocellular carcinoma; SKCM, skin cutaneous melanoma; THCA, thyroid carcinoma; MDSC, myeloid derived suppressor cells. *P* < 0.05 was considered statistically significant.

The tumor data obtained from TCGA were classified into low and high expression groups based on the level of CNDP1 expression (median). We conducted an analysis to investigate the impact of CNDP1 expression on patient prognosis across various types of cancer. Our findings from forest plots revealed that a low level of CNDP1 was associated with a better OS outcome specifically in uterine carcinosarcoma (UCS), KIRP, and KIRC, while high expression of CNDP1 was linked to poorer OS in THCA (Figure 7a). Patients with low CNDP1 expression had favorable DFS in UCS and SARC. Conversely, patients with high CNDP1 expression had unfavorable DFS in PRAD and PAAD (Figure 7b). Furthermore, low expression of CNDP1 was associated with worse DSS in UCS, KIRP, and KIRC, but high expression of CNDP1 was associated with THCA (Figure 7c). Low expression of CNDP1 indicated better PFI in UCS and KIRC, while high expression showed worse PFI in THYM (Figure 7d). Additionally, low expression of CNDP1 was linked to favorable DFI in UCS and SARC, while a high level of CNDP1 was associated with a worse DFI outcome for patients (Figure 7e).

We further explored the correlation between CNDP1 expression and clinical outcomes in eight cancers, where notable differences in CNDP1 levels between cancerous tissues and their normal counterparts were observed, employing OS analysis (Figure 7f–m). The survival analysis disclosed that in KIRC, the disparity in survival between the groups with high and low CNDP1 expression was statistically significant (*P* = 0.012), with patients exhibiting elevated CNDP1 levels experiencing superior OS compared to their low-expression counterparts. In LIHC (HCC), the survival curves of the two groups were markedly distinct, with those in the high CNDP1 expression group achieving greater OS during an 80-month observation period (*P* = 0.073). These findings imply that diminished CNDP1 expression correlates strongly with adverse prognoses in HCC patients, positioning CNDP1 as a potentially valuable prognostic biomarker in HCC.

We delved further into the correlation between the variation in CNDP1 expression and the prognostic outcomes in HCC patients across varying tumor microenvironments (Figure 7n–s). The findings indicated that diminished expression of CNDP1 was linked with an adverse prognosis in HCC cases. Moreover, elevated expression levels of CD4^+^ Th2 cells, mast cells, hematopoietic stem cells, and MDSC were associated with unfavorable prognostic implications for patients. *P* < 0.05 was considered statistically significant.

### 3.8 Prediction model for microvascular invasion (MVI)

**Figure 8:**
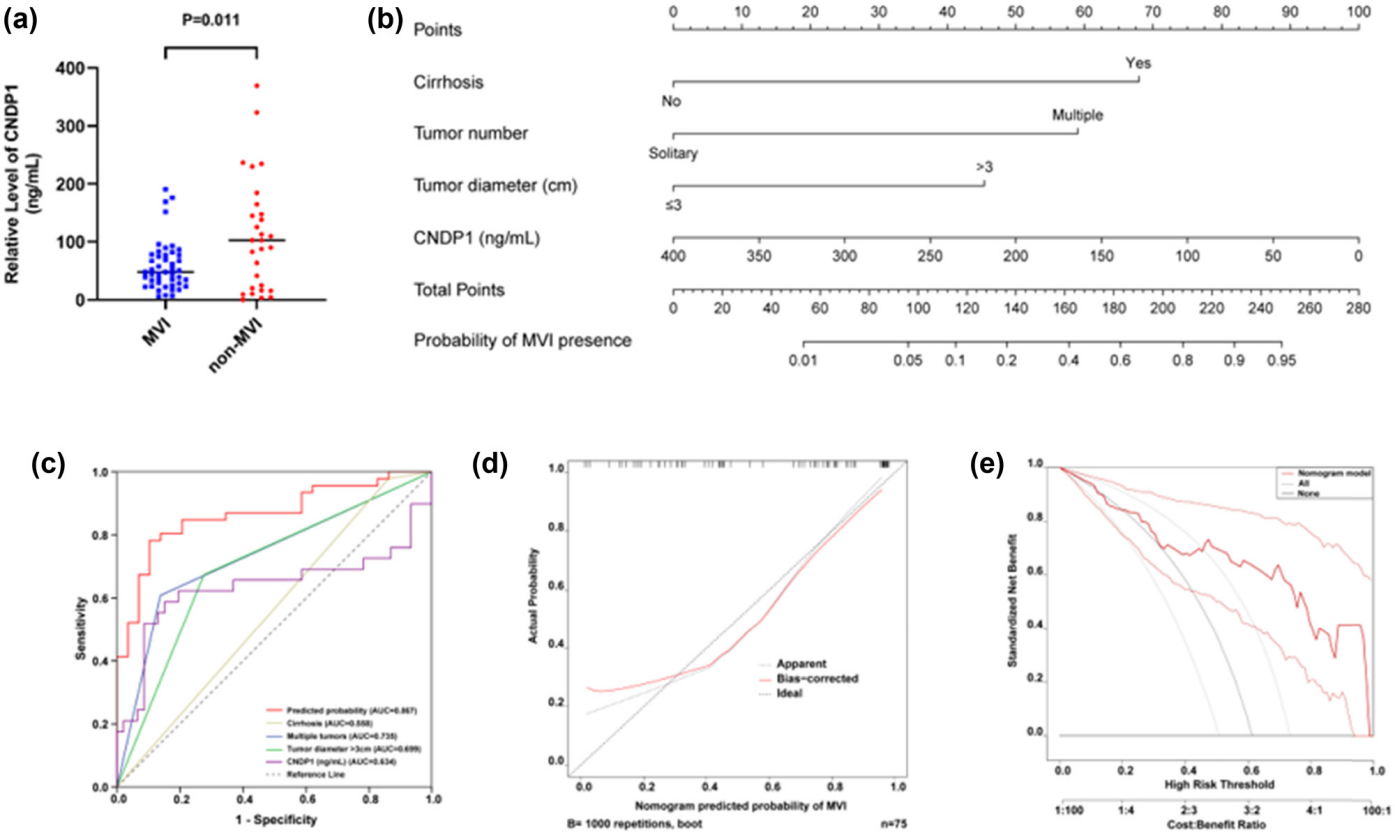
Prediction model to estimate the risk of MVI presence preoperatively in HCC. (a) Serum CNDP1 level in the MVI group exhibited a significant decrease. (b) Nomogram to estimate the risk of MVI presence preoperatively in HCC. The nomogram incorporates cirrhosis, tumor number, tumor diameter, and serum CNDP1 level. (c) ROC curve based on the prediction model and other indicators in the model (*n* = 75). (d) Calibration curve based on the prediction model (*n* = 75). (e) Decision curve based on the prediction model (*n* = 75). MVI, microvascular invasion; HCC, hepatocellular carcinoma; CNDP1, carnosine dipeptidase 1. *P* < 0.05 was considered statistically significant.

A nomogram was devised to forecast the likelihood of MVI, utilizing preoperative data. This model assessed tumor-specific parameters, including tumor size and count, in addition to the presence of liver cirrhosis, through preoperative diagnostic imaging. Serum samples were obtained from 75 HCC patients enrolled in this study, and the levels of serum CNDP1 were quantified by ELISA. The findings indicated a markedly lower level of serum CNDP1 in the MVI group compared to the non-MVI group (*P* = 0.011) (Figure 8a). Therefore, in this study, we incorporated CNDP1 into the subsequent stage of univariate logistic regression analysis to assess its prognostic significance for MVI in patients with HCC.

Univariate logistic analysis demonstrated significant associations between cirrhosis (*P* = 0.085), tumor number (*P* < 0.001), tumor size (*P* = 0.001), and serum CNDP1 level (*P* = 0.007) with MVI occurrence (Table 4). Multivariate logistic regression analysis incorporating the above four variables identified that cirrhosis (*P* = 0.050), multiple tumors (*P* = 0.002), tumor size (*P* = 0.007), and CNDP1 level (*P* = 0.021) were independent predictors of MVI (Table 5). Thus, the final logistic regression equation included these four factors: *Y* = 2.623 × cirrhosis + 2.278 × tumor number + 1.752 × tumor diameter − 0.010 × serum CNDP1 concentration − 2.789. A nomogram was subsequently constructed based on this model (Figure 8b), facilitating a visual prediction of the risk of MVI prior to surgical interventions in individuals diagnosed with HCC. This nomogram exhibited exemplary predictive accuracy for assessing the risk of MVI, evidenced by a concordance index of 0.867 [95% CI (0.784–0.949)] (Figure 8c). Calibration plots convincingly demonstrated a satisfactory concordance between the predicted risk by the nomogram and actual MVI estimates (Figure 8d). The decision curve associated with the nomogram is illustrated in Figure 8e.

**Table 4:** Univariate logistic analysis results of the patients

Variable	Univariable	
	OR (95% CI)	*P* value
Age (years)	0.973 (0.928–1.020)	0.257
Sex (male)	2.739 (0.701–10.709)	0.147
HBV infection	1.453 (0.435–4.855)	0.543
Blood ammonia (μmol/L)	0.999 (0.977–1.022)	0.940
PT (s)	0.896 (0.675–1.189)	0.445
WBCs (×10^9^/L)	0.883 (0.631–1.237)	0.471
RBCs (×10^12^/L)	1.108 (0.522–2.353)	0.789
Platelets (×10^9^/L)	0.998 (0.992–1.005)	0.629
AFP (ng/mL)	1.001 (0.998–1.004)	0.461
Albumin (g/L)	1.002 (0.929–1.082)	0.952
ALT (U/L)	1.002 (0.985–1.019)	0.836
Cirrhosis	7.200 (0.763–67.983)	0.085*
Tumor number, multiple vs solitary	9.772 (2.899–32.610)	<0.001*
Tumor diameter, >3 vs ≤3 (cm)	5.425 (1.954–15.065)	0.001*
CNDP1 (ng/mL)	0.989 (0.981–0.997)	0.007*

OR, odds ratio; CI, confidence interval; HBV, hepatitis B virus; PT, prothrombin time; WBCs, white blood cells; RBCs, red blood cells; AFP, alpha-fetoprotein; ALT, alanine aminotransferase; CNDP1, carnosine dipeptidase 1. *Indicates *P* < 0.1.

**Table 5:** Multivariate logistic analysis results of the patients

Variable	*β* ^a^	OR (95% CI)	*P* value
Cirrhosis	2.623	13.783 (1.002–189.687)	0.050*
Tumor number, multiple vs solitary	2.278	9.762 (2.301–41.422)	0.002*
Tumor diameter, >3 vs ≤3 (cm)	1.752	5.768 (1.617–20.573)	0.007*
CNDP1 (ng/mL)	-0.010	0.990 (0.982–0.999)	0.021*
Constant	-2.789	0.061	0.050

OR, odds ratio; CI, confidence interval; CNDP1, carnosine dipeptidase 1. ^a^Unstandardized *β* coefficients were calculated from the multivariate logistic regression model. *Indicates *P* < 0.05.

## 4 Discussion

Gene expression profiling using microarray technology serves as a quintessential instrument for demystifying the intrinsic mechanisms of diseases and pinpointing genes and pathways associated with various malignancies, which may have remained undiscovered. This methodology sheds light on the molecular underpinnings of cancer and delineates potential avenues for therapeutic endeavors [25]. In order to identify potential prognostic biomarkers associated with the development of pan-cancer, especially HCC, we conducted an analysis of genomic microarray data from GSE40367. Our scrutiny led to the identification of 2,248 DEGs, comprising 1,121 up-regulated and 1,127 down-regulated genes. Gene bioinformatics offers a potential molecular targeting approach for the prevention and management of HCC. We executed a comprehensive series of enrichment analyses including GO, KEGG, and GSEA. The findings suggest that the DEGs in GSE40367may be linked to pathways related to biological oxidations, cell cycle, and fatty acid degradation when compared to the control group. These results align with previous research studies [26–28].

In our literature search, we found no publications that have conducted pan-cancer analyses of CNDP1 from a comprehensive tumor perspective. Therefore, we conducted a thorough examination of the CNDP1 in pan-cancer based on data from TCGA, GEO, HPA, and UniProt databases. This examination included an analysis of molecular characteristics such as gene expression, promoter methylation, biological function, and protein structure. It was observed that CNDP1 is generally expressed at low levels in most tumors, including HCC. Additionally, it was noted that CNDP1 is both a secreted and intracellular protein located outside the cell membranes. It is recognized for its ability to catalyze the hydrolysis of Xaa-His dipeptide by peptide bonds.

Tumor-infiltrating immune cells, pivotal elements within the tumor microenvironment, are intimately linked with the genesis, progression, and dissemination of cancer [29,30]. Cancer-related fibroblasts located within the stromal region of the tumor microenvironment have been documented to play a role in influencing the activity of diverse immune cells that infiltrate the tumor [31]. In this research, we employed CIBERSORT, EPIC, and other algorithms to explore the potential interplay between varying levels of immune cell infiltration and CNDP1 expression across diverse cancer types in the TCGA. We discerned a statistically significant inverse relationship between B-cell immune infiltration, as determined by the EPIC algorithm, and CNDP1 expression in six distinct tumors, including THYM. Additionally, our scrutiny of immune cell infiltration in HCC disclosed a negative association between CNDP1 expression and the presence of B cells, CD4^+^ T cells, CD8^+^ T cells, T regulatory cells, DMSC, mast cells, myeloid dendritic cells, monocytes, and common lymphoid progenitor cells. These insights imply that CNDP1 may play a role in forecasting patient outcomes in HCC and other tumors. Specifically, elevated levels of CNDP1 expression might correlate with a more favorable prognosis for patients.

Deletion or duplication of genome fragments larger than 1 kb resulting from genome rearrangement is called CNV. Genome CNV is part of normal human genetic variation [32–34]. TMB is crucial as biomarkers in the prediction of tumor immunotherapy outcomes and functions as important immune regulatory elements [35]. DNA methylation, an epigenetic mechanism, profoundly influences gene transcription [36]. Consequently, in this study, we investigated the relationship between CNDP1 expression and CNV, TMB, and promoter methylation across various cancers. Our findings indicate that the interplay between CNV, TMB, promoter methylation, and CNDP1 expression differs across cancer types. In HCC, a negative correlation exists between CNV and TMB, while a positive correlation is observed between promoter methylation and CNDP1 expression. These observations suggest that the prognostic value of CNDP1 varies among different cancers, and elevated expression of CNDP1 in HCC may signify a more favorable prognosis.

However, survival prognostic analysis of the *CNDP1* resulted in different conclusions for different tumors. Our research revealed a notable correlation between reduced levels of *CNDP1* expression and unfavorable outcomes in patients with HCC. Moreover, elevated levels of CD4^+^ Th2 cells, mast cells, hematopoietic stem cells, and MDSC were correlated with an unfavorable prognosis in patients. These observations indicates that CNDP1 may act as a crucial prognostic biomarker in HCC.

Relevant research indicates that MVI serves as the initial stage in the progression of HCC, ultimately leading to intrahepatic tumor spreading or systemic metastases [37,38]. MVI has been acknowledged as a pivotal prognostic indicator subsequent to hepatic resection for HCC [39–42]. Following the outcomes of multivariate logistic regression analysis, we developed a logistic regression equation incorporating cirrhosis, tumor number, tumor diameter, and serum CNDP1 level. Subsequently, we constructed a nomogram for predicting MVI, which enhanced the interpretability and convenience of the prediction model for clinicians. The findings corroborate that CNDP1 serves as an independent predictor of MVI in patients with HCC.

## 5 Conclusion

In conclusion, our research demonstrates that CNDP1 is consistently underexpressed across various cancer types, including HCC. Moreover, diminished levels of CNDP1 correlate with adverse outcomes in these malignancies. Our analysis further disclosed that the enrichment pathway of DEGs in GSE40367, along with associated immune infiltration in pan-cancer, has been elucidated. These insights may enhance our comprehension of the molecular underpinnings and furnish clinically pertinent molecular targets for prognostication in both pan-cancer and HCC contexts. In essence, this study offers invaluable support in pinpointing critical prognostic biomarkers for both pan-cancer and HCC.
